# Post-translational modifications orchestrate mTOR-driven cell death in cardiovascular disease

**DOI:** 10.3389/fcvm.2025.1620669

**Published:** 2025-07-15

**Authors:** Jiawei Guo, Yiting Wu, Zhengdong Wan, Zhaoshan Zhang

**Affiliations:** ^1^ Department of Vascular and Endovascular Surgery, The First Affiliated Hospital of Yangtze University, Jingzhou, China; ^2^ Department of Pharmacology, School of Medicine, Yangtze University, Jingzhou, China; ^3^ Department of Radiology, Jingzhou Hospital Affiliated to Yangtze University, Jingzhou, Hubei, China

**Keywords:** MTOR signaling, protein modifications, cardiovascular diseases, cell death, therapeutic strategies

## Abstract

The mechanistic target of rapamycin (mTOR) signaling pathway is a central regulator of cellular physiology, modulating processes such as metabolism, protein synthesis, growth, and various forms of cell death. Increasing evidence has revealed that dysregulation of mTOR activity, often triggered or exacerbated by aberrant post-translational modifications (PTMs), contributes to the onset and progression of cardiovascular diseases (CVDs), including atherosclerosis, myocardial infarction, heart failure, and ischemia-reperfusion injury. PTMs such as phosphorylation, ubiquitination, SUMOylation, acetylation, and glycosylation alter mTOR's upstream regulators and downstream effectors, influencing the balance between apoptosis, autophagy, pyroptosis, and ferroptosis. These regulatory mechanisms provide a molecular basis for cell fate decisions during cardiovascular stress and injury. In this review, we systematically summarize recent advances in the understanding of PTM-mediated control of mTOR signaling, with a focus on cardiovascular pathophysiology. We also highlight emerging therapeutic strategies that target PTMs or the mTOR axis, including mTOR inhibitors, AMPK activators, proteasome blockers, and SUMOylation modulators, all of which show promise in preclinical or clinical settings. Understanding how PTMs fine-tune mTOR activity and cell death may pave the way for novel, targeted interventions in cardiovascular medicine and offer potential avenues for the development of precision therapies.

## 1 Introduction

Cardiovascular diseases (CVDs), including atherosclerosis, myocardial infarction, and aortic dissection (AD), remain the leading cause of mortality worldwide, posing a significant global health challenge (1, 2). These conditions arise from complex interactions among inflammation, oxidative stress, and cell death, which encompasses both non-programmed forms, such as necrosis, and various types of programmed cell death (PCD), including apoptosis, ferroptosis, pyroptosis, and autophagy-dependent cell death (3–5). While necrosis primarily contributes to acute injuries, PCD plays a pivotal role in chronic pathological remodeling and tissue degeneration (6). Moreover, recent studies highlight the interplay between cell death modalities in CVDs, mediated by shared mechanisms such as oxidative stress and inflammatory signaling (7, 8). For example, necrosis-induced release of damage-associated molecular patterns (DAMPs) can exacerbate inflammation, triggering ferroptosis or pyroptosis and amplifying cardiovascular damage (9, 10). These interconnected processes underscore the critical need for a comprehensive understanding of the regulatory pathways that govern cell death in cardiovascular pathophysiology.

The mammalian target of rapamycin (mTOR) signaling pathway, a master regulator of cellular growth, metabolism, and survival, has emerged as a pivotal modulator of cell death in CVDs (11, 12). In addition to its canonical roles, mTOR signaling is intricately regulated by post-translational protein modifications such as phosphorylation, ubiquitination, and SUMOylation (13, 14). These modifications serve as molecular switches, bridging upstream stimuli and downstream responses, including oxidative stress regulation and ferroptosis (15). Investigating these regulatory mechanisms not only deepens our understanding of CVD progression but also opens new avenues for therapeutic interventions.

By focusing on the interplay between mTOR signaling, post-translational protein modifications, and cell death, this review provides novel insights into the pathogenesis of CVDs. These findings emphasize the potential for targeting these pathways to mitigate oxidative stress and cell death, ultimately advancing the development of innovative strategies for CVD treatment and prevention.

## 2 The role of mTOR signaling in cardiovascular diseases

mTOR is a highly conserved serine/threonine kinase that plays a central role in regulating cellular processes such as growth, metabolism, proliferation, and survival (16, 17). It integrates signals from nutrients, energy status, and growth factors to modulate key cellular activities, thereby ensuring proper cellular function in response to environmental changes (18). mTOR operates through two distinct complexes, mTORC1 and mTORC2, each of which plays specific roles in cellular regulation (19).

### 2.1 mTOR function and regulation

**Figure 1:**
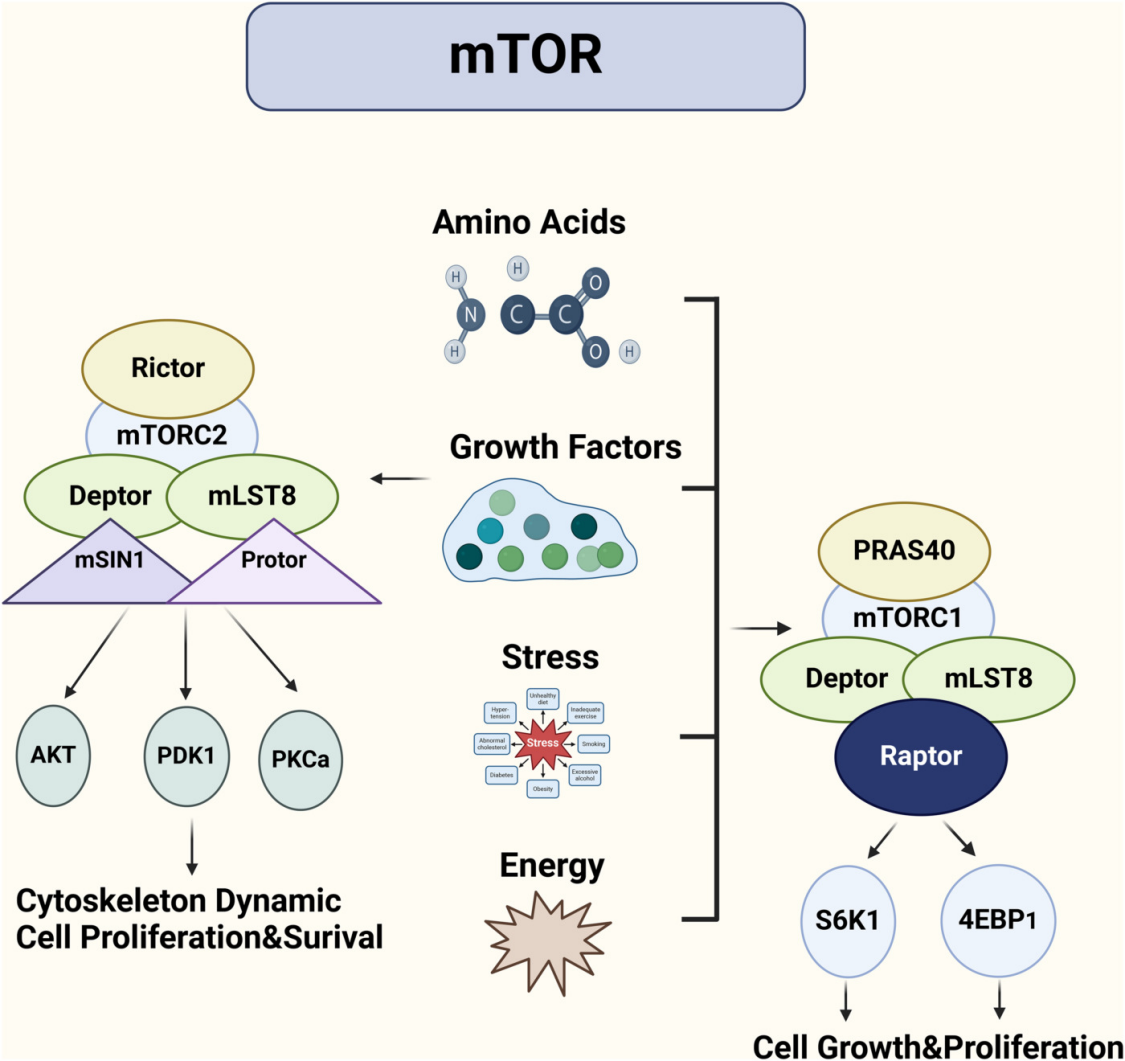
mTORC1 and mTORC2: distinct complexes regulating cell growth and survival in cardiovascular contexts. The mTOR signaling pathway regulates cell growth through two complexes: mTORC1 and mTORC2. mTORC1, acting as a highly sensitive sensor of growth factors and nutrients, is primarily responsible for promoting cell growth and proliferation. It activates protein synthesis by phosphorylating S6K and 4E-BP1. In contrast, mTORC2 is more involved in cell survival, metabolism, and cytoskeletal remodeling. By phosphorylating AKT/PKB, mTORC2 promotes cell survival and influences cell shape and movement.

mTORC1 is the more extensively studied of the two complexes and is a key regulator of cell growth and metabolism (Figure 1) (20, 21). It promotes protein synthesis by activating key effectors such as S6K1 (ribosomal protein S6 kinase 1) and 4EBP1 (eukaryotic translation initiation factor 4E-binding protein 1), which control translation initiation and protein synthesis. mTORC1 also regulates lipid biosynthesis and autophagy, coordinating these processes with nutrient availability (21–25). One of the key mechanisms by which mTORC1 exerts its effects is through the regulation of the AMPK (AMP-activated protein kinase) pathway, which responds to cellular energy levels (26, 27). In states of nutrient deficiency or stress, AMPK is activated and inhibits mTORC1, promoting energy conservation through autophagy (28, 29).

A study demonstrated the role of mTORC1 in muscle growth and metabolism, showing that mTORC1 activity is a critical determinant of protein synthesis in muscle cells (30). Furthermore, mTORC1 activity has been shown to be regulated by the availability of amino acids, particularly leucine, which acts as a signal for mTORC1 activation to promote cell growth and proliferation (31, 32). This has significant implications in contexts like cancer, where excessive mTORC1 activation contributes to uncontrolled cell proliferation (Figure 1) (33, 34).

mTORC2, in contrast to mTORC1, primarily regulates cell survival, cytoskeletal organization, and metabolism, and its role is less well understood (35, 36). One of the key functions of mTORC2 is the phosphorylation of Akt, a central regulator of cell survival and metabolism (37). Akt activation promotes glucose uptake, inhibits apoptosis, and supports cell proliferation. mTORC2 also regulates the actin cytoskeleton, influencing cell shape and motility, which is particularly important in cancer metastasis and tissue regeneration (38, 39).

A recent study emphasized the role of mTORC2 in endothelial cell function, showing that mTORC2 inhibition leads to a reduction in vascular remodeling, indicating its critical involvement in endothelial cell survival and function in vascular diseases (40).Additionally, mTORC2-mediated phosphorylation of Akt has been implicated in the development of atherosclerosis, where it contributes to endothelial dysfunction and vascular inflammation (41, 42).

### 2.2 mTOR in CVDs

The mechanistic target of rapamycin (mTOR) plays a multifaceted role in the pathogenesis of CVDs, influencing cellular processes such as growth, metabolism, proliferation, and survival (43). mTOR is involved in regulating oxidative stress, inflammation, cell death, and vascular remodeling, all of which are crucial in the development and progression of CVDs (44, 45). Its activity, however, varies between different cardiovascular conditions, and dysregulation of mTOR signaling is associated with several pathological processes, including atherosclerosis, myocardial infarction, heart failure, and coronary artery disease (46).

In atherosclerosis, mTOR activation drives several processes contributing to plaque formation and instability (47). It promotes the proliferation and migration of vascular smooth muscle cells (VSMCs), facilitating the formation of the fibrous cap and contributing to plaque expansion (48). mTOR also influences endothelial cell function, increasing endothelial permeability and inducing oxidative stress, which exacerbates vascular inflammation and promotes foam cell formation (44, 49). Inflammatory cytokines, such as TNF-α, and factors like advanced glycation end products (AGEs) also contribute to mTOR activation, further enhancing the inflammatory environment within plaques (50, 51). This persistent activation of mTOR accelerates the development of atherosclerosis, increases plaque vulnerability, and contributes to plaque rupture, a key event in the pathogenesis of acute coronary events (52).

mTOR's role in myocardial infarction (MI) and heart failure is similarly complex (53, 54). After MI, mTOR signaling is involved in myocardial hypertrophy, fibrosis, and tissue remodeling (55). It promotes the production of extracellular matrix proteins, such as collagen, and inhibits autophagy, a process critical for clearing damaged cellular components (56). This results in increased fibrosis and scarring, impairing the heart's ability to regenerate and repair itself (56). While mTOR activation can initially promote hypertrophic responses in the myocardium as an adaptive mechanism to stress, prolonged activation contributes to pathological remodeling, including ventricular dilation and heart failure (57, 58). Moreover, excessive mTOR signaling can exacerbate ischemic injury by promoting oxidative stress and inflammation, which leads to further tissue damage (59).

In coronary artery disease (CAD), mTOR signaling is integral to the regulation of endothelial function, vascular tone, and smooth muscle cell behavior (Table 1) (60). mTOR modulates the response to oxidative stress in endothelial cells, contributing to endothelial dysfunction, a key early event in CAD (61). mTOR also plays a role in VSMC proliferation and migration, which are important in the development of neointimal hyperplasia following vascular injury (62). In this regard, mTOR regulates the expression of matrix metalloproteinases (MMPs) and other enzymes involved in extracellular matrix remodeling, leading to changes in vascular wall structure and increased susceptibility to rupture in atherosclerotic lesions (63, 64). Furthermore, the activation of mTOR in the context of CAD is associated with an imbalance in the vascular response to stress and injury, ultimately promoting the progression of the disease (65).

**Table 1:** Functional differences between mTORC1 and mTORC2 in cardiovascular diseases.

Disease	mTORC1-mediated mechanism	mTORC2-mediated mechanism	References
Atherosclerosis	Promotes inflammation, increases ROS production, facilitates foam cell formation, accelerates plaque growth	Improves endothelial function, inhibits inflammation, reduces vascular damage	(66, 67)
Aortic dissection	Activates MMPs, promotes vascular wall degeneration, increases oxidative stress, leads to vascular structural instability	Promotes vascular repair, inhibits endothelial cell damage	(68, 69)
Myocardial infarction	Increases oxidative stress and cell death, exacerbates ischemia-reperfusion injury	Protects cardiomyocyte survival, improves heart function, reduces oxidative stress damage	(53)
Hypertension	Promotes vasoconstriction, increases vascular tension, induces endothelial dysfunction	Enhances endothelial relaxation function, reduces hypertension-induced vascular damage	(70, 71)
Heart failure	Activates fibrotic pathways, causes myocardial remodeling, increases energy metabolism dysregulation	Protects cardiomyocytes, regulates energy metabolism adaptation, alleviates cardiac burden	(72, 73)
Diabetes-related cardiovascular disease	Promotes lipid accumulation, exacerbates oxidative stress, increases the risk of arteriosclerosis	Improves glucose metabolism homeostasis, alleviates cardiovascular damage caused by diabetes	(74, 75)
Cardiac hypertrophy	Promotes protein synthesis, accelerates cardiomyocyte hypertrophy, increases oxidative stress	Regulates antioxidant mechanisms, reduces ROS accumulation in cells, inhibits cardiac hypertrophy	(76, 77)
Arrhythmia	Causes calcium overload, disrupts myocardial electrical activity stability, increases the risk of arrhythmia	Maintains intracellular calcium homeostasis, reduces oxidative stress-induced electrical activity abnormalities	(78, 79)

In heart failure, mTOR signaling also plays a pivotal role in the transition from compensatory hypertrophy to decompensated heart failure (Table 1) (80). Dysregulated mTOR activity has been implicated in the pathogenesis of heart failure with both reduced and preserved ejection fraction (HFrEF and HFpEF) (Table 1) (81, 82). In the failing heart, mTOR activation may increase oxidative stress, leading to mitochondrial dysfunction and contractile dysfunction (83, 84). mTOR's role in fibrosis, inflammation, and autophagy inhibition in the heart further accelerates cardiac remodeling, fibrosis, and loss of myocardial function (85).

Given mTOR's central role in the pathogenesis of these cardiovascular conditions, targeting this pathway holds significant therapeutic promise (Table 1) (86). However, mTOR functions as a complex hub that regulates multiple cellular processes, and its effects in CVDs are context-dependent (Table 1). While mTOR inhibition with rapamycin or other mTOR inhibitors has shown promise in preclinical models, clinical translation remains challenging due to the broad effects of mTOR on various cell types and tissues (87). Furthermore, as mTOR signaling has both protective and deleterious roles in different stages of CVDs, a more nuanced approach is required (88). Selective modulation of mTOR complexes (such as mTORC1 or mTORC2) or targeting downstream effectors may provide a more refined therapeutic strategy, potentially improving clinical outcomes by mitigating the adverse effects of excessive mTOR activation without compromising its beneficial roles in tissue repair and regeneration (Table 1).

In conclusion, mTOR is a pivotal regulator in the pathogenesis of CVDs, influencing numerous aspects of cardiovascular function (Table 1). A better understanding of the intricate mechanisms by which mTOR regulates oxidative stress, inflammation, and cell death will be critical for the development of targeted therapies (89). Tailoring mTOR inhibition or modulation to specific disease contexts, timing, and patient populations will be essential to maximize therapeutic benefits and minimize potential risks (90).

In order to better understand the distinct roles that mTORC1 and mTORC2 play in various cardiovascular diseases, the following table summarizes their respective mechanisms and effects across a range of conditions. This comparison highlights how mTOR signaling pathways contribute to disease progression and how differential activation of mTORC1 and mTORC2 could off.

## 3 Mechanisms underlying cell death in cardiovascular disease

**Figure 2:**
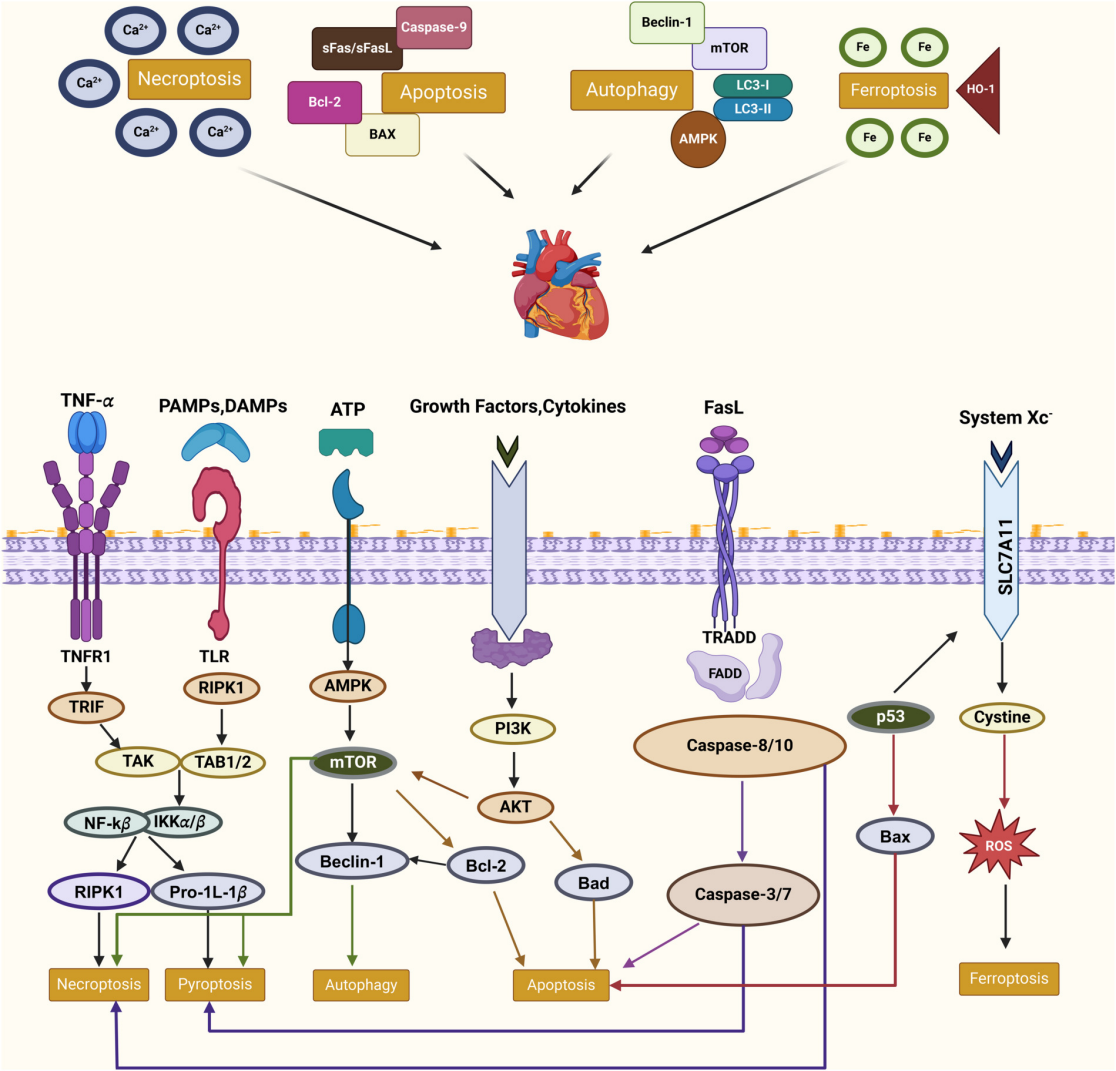
Molecular interplay of PCD pathways in cardiovascular diseases this figure illustrates the interconnected molecular mechanisms of PCD pathways—apoptosis, necroptosis, autophagy, and ferroptosis—in CVDs. Key regulators such as RIPK1, mTOR, and AMPK mediate these processes, influenced by external signals like TNF-α and oxidative stress. Crosstalk between pathways, including shared mediators like RIPK1 and the interplay of autophagy with apoptosis or ferroptosis, highlights their collective roles in inflammation, oxidative stress, and cell fate. These relationships underscore the therapeutic potential of targeting PCD in CVD management.

Cell death is a central pathological event in CVDs, contributing to the progression of conditions such as atherosclerosis, myocardial infarction, and heart failure (Figure 2) (91). The intricate interplay between programmed cell death (PCD) and non-programmed cell death (non-PCD) shapes the structural and functional outcomes of cardiovascular tissues (92, 93). Below, we detail the mechanisms underlying these two forms of cell death and their implications in CVD pathophysiology.

### 3.1 Programmed cell death in cardiovascular diseases

PCD refers to tightly regulated cellular processes involving molecular pathways that orchestrate cell demise (94). Its various forms—such as apoptosis, autophagy-dependent cell death, ferroptosis, and pyroptosis—play distinct but interconnected roles in cardiovascular pathology (95).

Apoptosis, a caspase-mediated process, is pivotal in both acute and chronic cardiovascular injury (96). In myocardial infarction, ischemia-reperfusion injury triggers mitochondrial dysfunction and cytochrome c release, activating apoptotic cascades (97).Similarly, in atherosclerosis, the apoptosis of vascular smooth muscle cells weakens plaque stability, predisposing it to rupture and thrombosis (98).

Autophagy is primarily a survival mechanism, but excessive autophagy contributes to cell death under stress conditions, such as hypoxia or oxidative stress (99). In heart failure, dysregulated autophagy exacerbates cardiomyocyte loss and disrupts myocardial contractility, while in vascular diseases, impaired autophagic clearance leads to foam cell accumulation and plaque progression (100, 101).

Iron-dependent lipid peroxidation characterizes ferroptosis, which is distinct from other PCD modalities (102). This form of cell death is implicated in oxidative stress-driven endothelial dysfunction and vascular remodeling (103). For instance, ferroptosis accelerates smooth muscle cell depletion in aortic dissection, compromising vessel integrity and facilitating aneurysm formation (104).

Gasdermin-mediated pyroptosis serves as a pivotal nexus between cell death and inflammation (105). Activation of the NLRP3 inflammasome in macrophages triggers pyroptosis, leading to the release of pro-inflammatory cytokines and subsequent destabilization of atherosclerotic plaques (106). In the context of heart failure, pyroptosis amplifies myocardial inflammation, thereby exacerbating pathological cardiac remodeling (107).

### 3.2 Non-programmed cell death in cardiovascular diseases

Non-PCD encompasses unregulated, passive forms of cell death, including necrosis and lysosome-mediated cell death, which are frequently observed in acute tissue damage associated with CVDs (Table 2) (108).

**Table 2:** Classification and pathophysiological roles of cell death mechanisms in cardiovascular diseases.

Type of cell death	Subtype	Key mechanisms	Role in CVDs	References
PCD	Apoptosis	Activation of caspases, release of cytochrome c from mitochondria, DNA fragmentation	Promotes cardiomyocyte apoptosis, endothelial dysfunction, and plaque destabilization	(109)
	Autophagy-dependent death	Excessive autophagosome activation leading to lysosomal dysfunction	Loss of cardiomyocytes, endothelial dysfunction, and foam cell accumulation	(110)
	Ferroptosis	Iron-dependent lipid peroxidation and inactivation of glutathione peroxidase 4 (GPX4)	Loss of smooth muscle cells, vascular wall instability, and aggravated arterial remodeling	(111)
	Pyroptosis	Activation of inflammasomes and gasdermin-mediated membrane pore formation	Release of inflammatory cytokines, destabilization of atherosclerotic plaques, and amplification of myocardial inflammation	(112)
Non-PCD	Necrosis	Cell membrane rupture and release of DAMPs, triggering secondary inflammation	Amplifies inflammatory responses, forms necrotic cores, and increases plaque rupture risk	(113)
	Lysosome-mediated death	Lysosomal membrane permeabilization and release of cathepsins into the cytoplasm	Increases proteotoxic stress, oxidative stress, and cardiomyocyte loss	(114)

Necrosis is characterized by membrane rupture and the uncontrolled release of intracellular contents, leading to secondary inflammation (115). In myocardial infarction, massive necrosis induced by prolonged ischemia activates inflammatory cascades, aggravating cardiac dysfunction (116). Additionally, necrotic core formation within atherosclerotic plaques promotes plaque instability, increasing the risk of rupture (Table 2) (117).

Lysosomal membrane permeabilization releases cathepsins and other hydrolytic enzymes into the cytosol, initiating cell death (118). In diabetic cardiomyopathy, lysosomal dysfunction exacerbates oxidative stress and lipid accumulation, driving myocardial damage (119). Similarly, in heart failure, impaired lysosomal clearance of damaged organelles contributes to proteotoxic stress and cardiomyocyte loss (120).

### 3.3 Synergy between programmed and non-programmed cell death

The interactions between PCD and non-PCD amplify cardiovascular damage, creating a feedback loop of inflammation and oxidative stress (121). For example, necrosis-derived damage-associated molecular patterns (DAMPs) not only exacerbate inflammatory responses but also trigger ferroptosis and pyroptosis, amplifying endothelial and myocardial injury (122, 123). Similarly, pyroptosis-induced cytokine release can aggravate necrotic cell death in vascular lesions, perpetuating plaque vulnerability (Table 2) (124).

The dichotomy and interplay between PCD and non-PCD underscore the complexity of cell death mechanisms in CVDs. Understanding these processes at a molecular level offers opportunities for developing therapeutic strategies to mitigate cardiovascular damage by targeting specific forms of cell death. Future research focusing on the crosstalk between these pathways may pave the way for novel, integrative treatments for cardiovascular diseases (Table 2). To better illustrate these mechanisms and their respective roles in CVDs, the following table provides a structured overview of both PCD and NPCD, emphasizing their pathophysiological implications in various cardiovascular conditions.

## 4 The relationship between mTOR and cell death in cardiovascular diseases

The connection between mTOR and cell death pathways is vital for understanding the mechanisms underlying cardiovascular pathophysiology and for identifying potential therapeutic targets (125). This section explores how mTOR modulates both types of cell death and their implications in CVDs.

### 4.1 mTOR and programmed cell death: mechanisms and implications

Programmed cell death (PCD) refers to a controlled process by which cells undergo death in response to specific signals (126). It includes various forms, such as apoptosis, autophagy, ferroptosis, and pyroptosis, each of which plays distinct roles in cardiovascular pathophysiology (127). mTOR is a key player in regulating PCD, acting as a switch that can either promote or inhibit these processes depending on the cellular context.

#### 4.1.1 mTOR and apoptosis

mTOR influences apoptosis through several downstream targets, including the pro-survival factors such as Bcl-2 and anti-apoptotic proteins (128). Dysregulation of mTOR can lead to the excessive apoptosis of endothelial cells, VSMCs, and cardiomyocytes, contributing to the progression of diseases such as atherosclerosis, myocardial infarction, and aortic dissection (129). In atherosclerosis, ubiquitination of mTORC1 components by E3 ligases such as FBXW7 has been shown to regulate macrophage apoptosis, influencing plaque stability. This highlights the role of mTORC1 ubiquitination in disease progression. In atherosclerosis, ubiquitination of mTORC1 components by E3 ligases such as FBXW7 has been shown to regulate macrophage apoptosis, influencing plaque stability (130). This highlights the role of mTORC1 ubiquitination in disease progression.

#### 4.1.2 mTOR and autophagy

Autophagy, a cellular process that removes damaged organelles and proteins, is another PCD process regulated by mTOR (131). mTOR inhibition promotes autophagy, which may help clear damaged components in the cardiovascular system (132). However, excessive autophagy can also be detrimental, leading to cell death and contributing to vascular remodeling and heart failure (133). During ischemia-reperfusion injury, acetylation of Raptor modulates mTORC1 activity, thereby regulating cardiomyocyte autophagy and reducing necrotic cell death (134). This PTM acts as a critical switch under oxidative stress.

#### 4.1.3 mTOR and ferroptosis

Ferroptosis, a recently identified form of iron-dependent cell death, is regulated by mTOR in certain cardiovascular diseases (135). mTOR-mediated signaling pathways, including those regulating oxidative stress and iron homeostasis, can influence the initiation of ferroptosis, which has been implicated in diseases like atherosclerosis and ischemic heart disease (136).

#### 4.1.4 mTOR and pyroptosis

Pyroptosis, a form of inflammatory cell death, is another PCD type regulated by mTOR (137). Inflammatory cytokines and ROS production, both modulated by mTOR, can drive pyroptosis in vascular cells, contributing to the inflammation and vascular damage seen in CVDs (138). In heart failure models, SUMOylation of mTORC2-associated proteins influences inflammasome activation and pyroptosis, contributing to myocardial inflammation and dysfunction (139).

### 4.2 mTOR and non-programmed cell death: role in cardiovascular pathology

Non-programmed cell death, primarily represented by necrosis, is characterized by uncontrolled cell rupture and inflammation (126). While less regulated than PCD, necrosis is a significant driver of acute tissue injury in cardiovascular diseases, and mTOR plays a role in its regulation (140).

#### 4.2.1 mTOR and necrosis

mTOR's role in necrosis is complex and context-dependent. Under conditions of excessive oxidative stress, such as ischemia-reperfusion injury, mTOR activation may contribute to the necrotic death of endothelial cells, VSMCs, and cardiomyocytes (141). This necrosis exacerbates tissue injury and promotes inflammation, creating a cycle of further cell damage.

#### 4.2.2 mTOR and inflammation

mTOR also regulates the inflammatory response, which is a critical mediator of necrosis. In the context of CVDs, mTOR activation in immune cells like macrophages and neutrophils can lead to the release of pro-inflammatory cytokines, amplifying the tissue damage and necrosis seen in conditions like myocardial infarction and aortic dissection (142).

In summary, mTOR plays a dual role in both programmed and non-programmed cell death in cardiovascular diseases. The pathways it regulates can either protect against cell death and promote tissue repair or exacerbate cell loss and inflammation. Understanding the intricate relationship between mTOR and cell death mechanisms is crucial for developing novel therapeutic strategies targeting mTOR signaling in cardiovascular diseases.

## 5 Protein modifications: the bridge between mTOR and cell death

**Figure 3:**
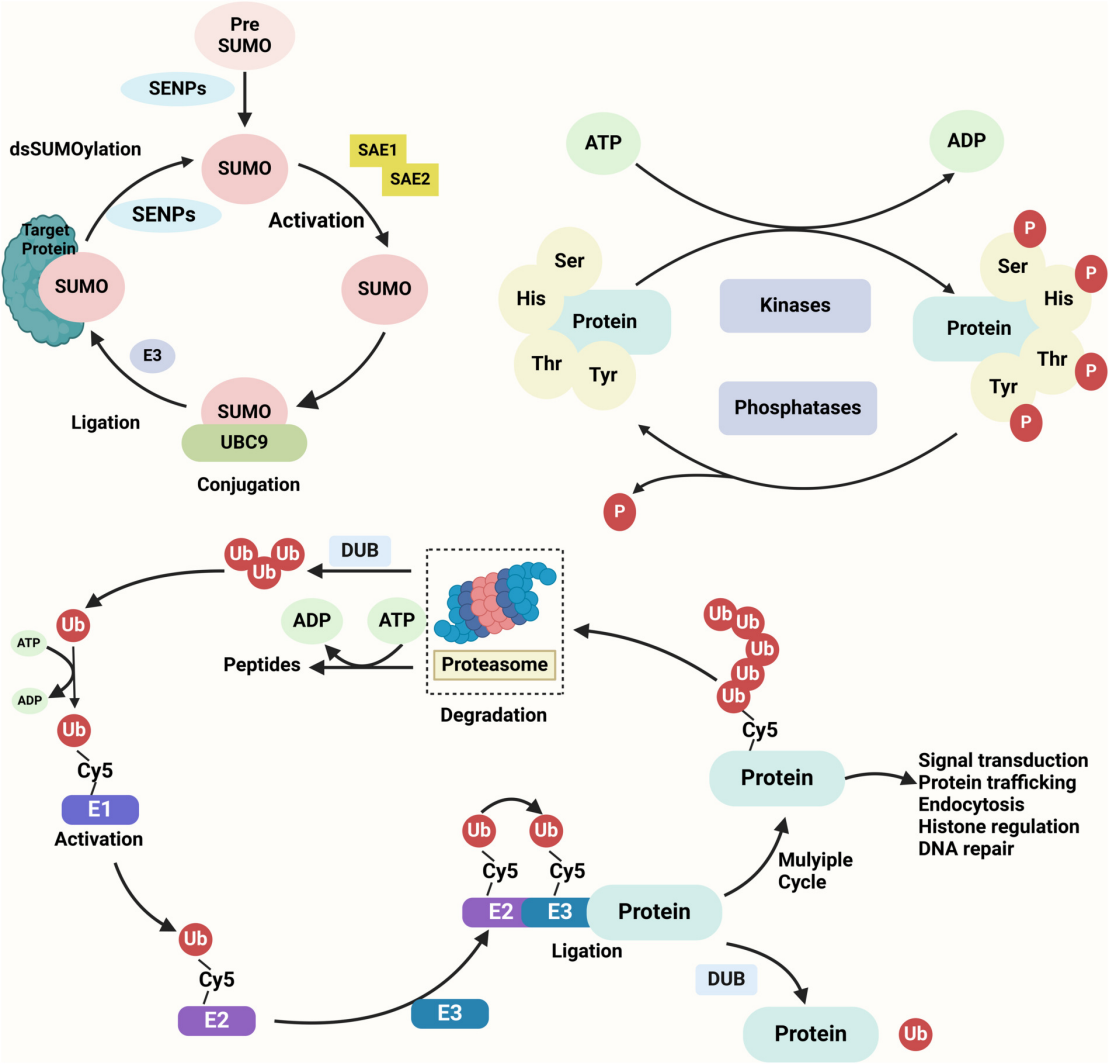
Key protein modifications in cellular regulation SUMOylation, phosphorylation, and ubiquitination are three essential post-translational modifications regulating protein function. SUMOylation involves the covalent attachment of SUMO to lysine residues on target proteins through a cascade of E1 activation, E2 conjugation, and E3 ligation, modulating processes such as nuclear transport, protein stability, and transcription. Phosphorylation, catalyzed by kinases, adds phosphate groups to serine, threonine, or tyrosine residues, influencing signaling pathways and cellular functions. Ubiquitination, mediated by E1, E2, and E3 enzymes, attaches ubiquitin to lysine residues, marking proteins for proteasomal degradation or altering their cellular roles. These modifications are reversible and tightly regulated, playing pivotal roles in cell cycle, apoptosis, and stress responses.

PMs play a pivotal role in linking mTOR signaling to cell death pathways, serving as a dynamic regulatory bridge in CVDs (Figure 3) (143). These modifications, including phosphorylation, ubiquitination, SUMOylation, acetylation, and glycosylation, finely regulate the activity, stability, and interactions of mTOR components (144, 145). Dysregulation of these modifications can trigger maladaptive responses, such as apoptosis, ferroptosis, and pyroptosis, influencing disease progression (Figure 3) (146, 147). Understanding the interplay between PMs and mTOR signaling offers novel therapeutic strategies to modulate cell fate and improve cardiovascular outcomes.

### 5.1 Protein modifications as critical regulators of mTOR signaling and cell death pathways in cardiovascular diseases

PMs represent a critical mechanism linking mTOR signaling to cell death pathways, serving as a dynamic regulatory bridge in CVDs (148). These modifications, including phosphorylation, ubiquitination, SUMOylation, acetylation, and glycosylation, orchestrate the balance between cell survival and death by modulating the activity, stability, and interactions of mTOR signaling components (149, 150).

Phosphorylation is one of the most extensively studied PMs in mTOR regulation (151). It enhances mTOR activity under physiological conditions but, when dysregulated, can trigger maladaptive responses such as apoptosis, ferroptosis, or pyroptosis (Table 3) (152). For instance, hyper-phosphorylation of mTOR and its downstream targets, such as p70S6 K or 4E-BP1, contributes to oxidative stress and cell death during ischemia-reperfusion injury (153, 154). Conversely, targeted inhibition of aberrant phosphorylation has been shown to mitigate myocardial damage in preclinical models (155).

**Table 3:** Regulatory mechanisms of protein modifications linking mTOR signaling and cell death in cardiovascular diseases.

Protein modification	Protein modification	Regulation of cell death	References
Phosphorylation	Activates mTORC1, inhibits mTORC2	Apoptosis Autophagy Necroptosis	(156)
Ubiquitination	Modulates mTOR stability and activity	Apoptosis Pyroptosis Ferroptosis	(157)
SUMOylation	Inhibits mTORC1, promotes autophagy	Inhibits apoptosis Autophagy	(158)
Acetylation	Activates mTORC1, inhibits mTORC2	Apoptosis Ferroptosis Necrosis	(159)
Methylation	Modulates mTORC1 activity	Apoptosis Autophagy	(160)
Glycosylation	Affects mTORC1 localization	Regulates apoptosis necrosis	(161)
Palmitoylation	Regulates mTORC1 and mTORC2 activity	Cell survival and death	(162)

Ubiquitination also plays a pivotal role in regulating mTOR signaling and cell death (Table 3) (163). K48-linked ubiquitination typically mediates proteasomal degradation, thereby suppressing mTOR activity, while K63-linked ubiquitination stabilizes the mTOR complex, promoting survival signals (164). Dysregulation of ubiquitination in conditions such as atherosclerosis exacerbates foam cell formation and apoptosis, destabilizing plaques and worsening disease progression (Table 3) (165, 166).

SUMOylation, a reversible PM involving the attachment of small ubiquitin-like modifiers, fine-tunes the activity of mTOR pathway components such as Raptor and TSC2 (167, 168). This modification can either promote autophagy during early stress adaptation or facilitate apoptosis under sustained oxidative stress (Table 3) (169). In VSMCs, SUMOylation influences phenotypic switching, playing a dual role in vascular remodeling and disease progression, as seen in aortic dissection and hypertension (170, 171).

Acetylation, another critical PM, regulates mTOR-mediated mitochondrial function and cardiomyocyte survival (Table 3) (172). For example, the acetylation status of mTOR targets can alter energy metabolism and redox balance during heart failure, while deacetylation therapies targeting the SIRT1-mTOR axis have shown potential in alleviating diabetic cardiomyopathy (173, 174).

Among the various PTMs involved in cardiovascular regulation of mTOR signaling, ubiquitination and acetylation are particularly well-characterized (175). Ubiquitination, mediated by E1 activating enzymes, E2 conjugating enzymes, and E3 ligases (such as FBXW7 and TRAF6), regulates mTOR complex stability and degradation (176, 177). K48-linked ubiquitination often targets mTORC1 components for proteasomal degradation, especially under stress conditions such as hypoxia or oxidative injury, leading to autophagy activation (178). In contrast, K63-linked ubiquitination may promote mTOR signaling under inflammatory conditions (179). Deubiquitinases (e.g., USP9X, OTUD7B) can stabilize mTOR and suppress cell death pathways (180, 181). Acetylation, catalyzed by enzymes such as p300/CBP or GCN5, modulates mTOR signaling by modifying upstream regulators (e.g., TSC2) or mTORC1 components (e.g., Raptor) (182). For instance, stress-induced acetylation of Raptor has been shown to enhance its interaction with mTOR, thereby suppressing autophagy (183). Conversely, deacetylases like SIRT1 can promote autophagy and inhibit apoptosis by deacetylating Atg proteins or FoxO transcription factors (184). These modifications often occur in a stimulus- and context-dependent manner, serving as rapid switches in the cellular response to cardiovascular injury.

These diverse PMs not only regulate mTOR activity but also mediate its downstream impact on cell fate, highlighting their therapeutic potential (185, 186). Pharmacological modulation of specific PMs has emerged as a promising approach to mitigate CVD progression (187). Drugs such as rapamycin derivatives (targeting phosphorylation), proteasome inhibitors (modulating ubiquitination), and SUMOylation inhibitors (e.g., TAK-981) have shown preclinical efficacy in restoring mTOR homeostasis and reducing pathological cell death (188).

In summary, protein modifications serve as a crucial bridge between mTOR signaling and cell death mechanisms, offering novel insights into the pathophysiology of CVDs. By targeting these modifications, innovative therapies could be developed to finely tune mTOR activity and improve cardiovascular outcomes.

### 5.2 Cross-talk between protein modifications in mTOR-dependent cell death

The intricate interplay between PMs represents a fundamental mechanism in regulating mTOR-dependent cell death pathways (147). These modifications, including phosphorylation, ubiquitination, and SUMOylation, rarely act in isolation; instead, they form a dynamic network of cross-talk that orchestrates mTOR activity and downstream signaling (189). By influencing the stability, localization, and functional output of mTOR and its associated proteins, this cross-regulation ensures cellular adaptation to environmental stresses (190). Disruptions in this delicate balance, however, can drive pathological cell death processes, such as apoptosis and necrosis, contributing to the progression of CVDs (191, 192). Exploring the cooperative and antagonistic interactions among PMs unveils new therapeutic possibilities for targeting mTOR in CVDs.

Notably, several PTMs regulating mTOR also impact other key signaling pathways involved in cardiovascular pathogenesis (193). For example, K63-linked ubiquitination of TRAF6 can activate both mTORC1 and NF-*κ*B signaling under inflammatory stress (194). Similarly, acetylation of STAT3 and mTOR by p300 under oxidative conditions promotes pro-hypertrophic gene expression and suppresses autophagy (195). These shared modifications reflect a level of multi-pathway integration in which PTMs serve as central regulatory nodes, coordinating diverse cellular outcomes in cardiovascular injury.

#### 5.2.1 Phosphorylation and ubiquitination cross-talk in mTOR-dependent cell death

Phosphorylation and ubiquitination are two of the most common protein modifications in cells, and they often interact with each other to regulate cell growth, division, and death (196, 197). Phosphorylation regulates protein function by activating or inhibiting specific kinases and phosphatases, while ubiquitination controls the stability and activity of proteins by marking them for degradation (198, 199).

The activation and function of mTOR are largely regulated by phosphorylation by upstream kinases such as AMPK and Akt (200, 201). The activity of these kinases is often closely linked to the ubiquitination process (202). For example, some proteins may be marked for ubiquitination and degradation following phosphorylation, or their half-life may be prolonged by the removal of ubiquitin (169). Activation of mTORC1 is associated with the phosphorylation of specific substrates (such as p70S6 K, 4EBP1), and the stability of these substrates may be influenced by ubiquitination (203, 204). Under stress conditions (e.g., oxidative stress or hypoxia), mTORC1 may regulate the ubiquitination of these substrates to control the balance between cell growth and death (205, 206).

Ubiquitination not only regulates the stability of mTOR downstream effectors but also participates in feedback regulation by controlling the activity of mTOR itself (207). By regulating the deubiquitination process of mTOR, the cell can precisely control the activation state of mTOR (208). During cell death, excessive activation of mTOR may lead to metabolic imbalances, while moderate ubiquitination modification can limit mTOR activity by promoting the degradation of mTOR complexes, preventing the cell from entering a state of overgrowth or metabolic imbalance (209, 210).

#### 5.2.2 SUMOylation and phosphorylation cross-talk in mTOR-mediated cell death

SUMOylation and phosphorylation are two important protein modifications in cells, with SUMOylation mainly regulating protein localization, stability, and function, while phosphorylation directly affects protein activity and interactions with other molecules (211–213).

SUMOylation can regulate the assembly and stability of mTOR, affecting its interactions with upstream regulators (such as Raptor) and downstream effectors (such as S6 K, 4EBP1) (214–216). For example, SUMOylation can increase the stability of Raptor, promoting mTORC1 activation, thus supporting cell growth and metabolism (217–219). During stress responses or drug treatment, SUMOylation and phosphorylation together regulate the activity of mTOR and influence cell death decisions (220). SUMOylation can also indirectly affect autophagy by regulating proteins associated with autophagy (such as LC3, Atg5) (221, 222).

In many cases, phosphorylation and SUMOylation occur in alternating modifications on the same protein, mutually regulating each other (223, 224). For example, under certain conditions, key regulatory proteins in the mTOR pathway may be activated by phosphorylation and then modulated by SUMOylation to promote cell growth and metabolism (225). In other cases, the introduction of SUMOylation may alter the output of phosphorylation responses, thereby affecting the cell death mechanisms (226, 227). The cross-regulation between SUMOylation and phosphorylation in mTOR-mediated cell death could determine whether cells enter apoptotic, necrotic, or autophagic death, especially in the context of cardiovascular diseases like myocardial ischemia or atherosclerosis (169, 228).

#### 5.2.3 Ubiquitination and SUMOylation in mTOR-mediated cell death

Ubiquitination and SUMOylation are both critical protein modification mechanisms within the cell, and they have a complex interaction (229, 230). While each modification mechanism acts independently, they often work in concert to regulate the mTOR pathway and cell death.

Ubiquitination and SUMOylation not only independently play roles in mTOR signaling, but they also influence each other, thus regulating protein function (231). For example, certain key regulatory proteins in the mTOR pathway (such as TSC2, Rheb) may be regulated through multiple modifications, including phosphorylation, ubiquitination, and SUMOylation, affecting mTOR activation (232–234). Under stress conditions, SUMOylation may prevent ubiquitination-mediated degradation of certain proteins by altering their localization, while ubiquitination may remove SUMO modifications, thereby changing the function of the protein (235).

In cardiovascular diseases, the cross-talk between ubiquitination and SUMOylation may regulate mTOR's stability and activity, controlling the growth and death of cells such as cardiomyocytes, endothelial cells, and smooth muscle cells (236). For example, in pathological conditions like atherosclerosis and myocardial infarction, these protein modifications may determine whether cells enter apoptosis, necrosis, or autophagy (237, 238).

## 6 Therapeutic potential of targeting protein modifications in mTOR-dependent cell death

PMs such as phosphorylation, ubiquitination, SUMOylation, and acetylation play pivotal roles in mTOR-dependent cell death pathways, offering potential therapeutic avenues for CVDs (239). This section discusses how targeting PMs can address specific disease mechanisms, with a focus on detailed disease contexts, pharmacological interventions, and emerging therapeutic approaches.

### 6.1 Targeting protein modifications in cardiovascular diseases

By influencing mTOR activity and its downstream signaling, specific PMs such as phosphorylation, ubiquitination, SUMOylation, and acetylation orchestrate the balance between cell survival and death (240). Dysregulation of these processes contributes to various CVDs, including atherosclerosis, myocardial infarction, heart failure, diabetic cardiomyopathy, and hypertension (241, 242).

In atherosclerosis, hyperactivation of the mTOR pathway exacerbates oxidative stress and foam cell apoptosis, destabilizing plaques and promoting vascular inflammation (243, 244). Phosphorylation of mTOR downstream effectors like p70S6 K and 4E-BP1 is often aberrant, driving endothelial dysfunction and VSMC phenotypic switching (245, 246). Concurrently, ubiquitination dysregulation modulates foam cell formation and inflammatory responses (247). Therapeutically, rapamycin and its derivatives, such as everolimus, mitigate these effects by attenuating mTOR phosphorylation (248).Proteasome inhibitors like bortezomib adjust the ubiquitination machinery, reducing foam cell apoptosis and stabilizing plaques (249, 250). These interventions highlight the potential of targeting mTOR-associated PMs to address the underlying pathology of atherosclerosis.

In ischemia-reperfusion injury (IRI) and myocardial infarction, the role of mTOR in cell death becomes evident through its modulation of apoptosis, necrosis, and ferroptosis (251, 252). SUMOylation of TSC2 during ischemia enhances mTOR activity, worsening oxidative stress and apoptotic signaling (Table 4) (253). Upon reperfusion, excessive phosphorylation of mTOR downstream proteins exacerbates inflammation, promoting cell death and myocardial dysfunction (254). Moreover, acetylation abnormalities impair mitochondrial function and energy metabolism, aggravating injury (Table 4) (255, 256). Interventions such as metformin, which activates AMPK and indirectly suppresses mTOR phosphorylation, have demonstrated cardioprotective effects by reducing apoptosis (257, 258). SUMOylation inhibitors like TAK-981 attenuate oxidative stress and protect mitochondrial integrity, while SIRT1 activators, including resveratrol, restore acetylation balance and metabolic homeostasis, reducing infarct size in preclinical studies (259, 260).

**Table 4:** Key protein modifications in mTOR-driven cardiovascular pathologies.

Cardiovascular disease	Protein modification	Mechanism	Therapeutic strategy	References
Atherosclerosis	Phosphorylation Ubiquitination	mTOR hyperactivation → oxidative stress, foam cell apoptosis, plaque instability	Rapamycin derivatives (e.g., everolimus), proteasome inhibitors (e.g., bortezomib)	(261)
Ischemia-reperfusion injury	SUMOylation Phosphorylation Acetylation	SUMOylation of TSC2 → mTOR activation, oxidative stress; acetylation imbalance → mitochondrial dysfunction	Metformin, SUMOylation inhibitors (e.g., TAK-981), SIRT1 activators (e.g., resveratrol)	(262)
Myocardial infarction	Phosphorylation Acetylation	Hyper-phosphorylation of mTOR effectors → apoptosis and inflammation	AMPK activators (e.g., metformin), Acetylation modulators	(263)
Heart failure	Phosphorylation Ubiquitination Acetylation	mTOR hyperactivation → hypertrophy, fibrosis; Ubiquitination dysregulation → inflammation	Rapamycin derivatives, Proteasome inhibitors, Acetylation modulators (e.g., resveratrol)	(264)
Diabetic cardiomyopathy	Ubiquitination Acetylation	Hyperglycemia → ubiquitination dysregulation, oxidative stress; Acetylation imbalance → inflammation	HDAC inhibitors (e.g., vorinostat), AMPK activators (e.g., metformin)	(265)
Hypertension	SUMOylation Phosphorylation	SUMOylation of VSMCs → phenotypic switching; mTOR hyperphosphorylation → endothelial dysfunction	SUMOylation inhibitors, Rapamycin derivatives	(266)

In chronic heart failure, aberrant PMs exacerbate pathological remodeling and cell death (Table 4) (267). Persistent mTOR overactivation, driven by excessive phosphorylation, leads to maladaptive cardiac hypertrophy and fibrosis (268). Ubiquitination dysregulation amplifies inflammatory cascades, while impaired acetylation compromises mitochondrial function and energy production (269). Rapamycin derivatives alleviate fibrosis by suppressing mTOR phosphorylation, whereas proteasome inhibitors adjust ubiquitination to curb inflammation and cell death (270). Additionally, acetylation modulators such as resveratrol enhance mitochondrial function, mitigating heart failure progression (271, 272).

Diabetic cardiomyopathy (DCM) exemplifies the interplay between metabolic derangements and PM dysregulation (Table 4) (273).In hyperglycemic conditions, disrupted ubiquitination destabilizes mTOR complexes, leading to heightened oxidative stress and apoptosis (274). Simultaneously, acetylation imbalance undermines mitochondrial function, exacerbating inflammation and fibrosis (275). Therapeutic agents like HDAC inhibitors (e.g., vorinostat) restore acetylation homeostasis and mitochondrial function, reducing inflammation in diabetic hearts (276). AMPK activators, including metformin, indirectly suppress mTOR hyperactivation and alleviate metabolic stress, improving cardiac outcomes in DCM models (Table 4) (277, 278).

Hypertension-induced vascular remodeling is another context in which PMs and mTOR dysregulation converge (Table 4) (279).Excessive SUMOylation in VSMCs drives their phenotypic switching, contributing to vascular stiffening and thickening (280). Additionally, hyperphosphorylation of mTOR effectors exacerbates endothelial dysfunction, increasing oxidative stress and apoptosis (281). Therapeutic strategies targeting these modifications, such as SUMOylation inhibitors to prevent VSMC phenotypic changes and rapamycin derivatives to curb mTOR hyperphosphorylation, show promise in mitigating vascular remodeling and improving vascular health (282, 283).

In summary, the intricate interplay between PMs, mTOR signaling, and cell death mechanisms highlights a promising therapeutic avenue for combating CVDs. Targeting specific PMs not only restores mTOR homeostasis but also addresses the underlying pathophysiological processes driving disease progression. Future research focusing on the development of PM-modulating drugs may unlock novel strategies to improve cardiovascular outcomes.

### 6.2 Targeted pharmacological strategies for mTOR-related protein modifications in cardiovascular diseases

The growing understanding of protein modifications and mTOR signaling in cardiovascular diseases has paved the way for the development of targeted therapies (Table 5). Rapamycin and its analogs have demonstrated efficacy in reducing atherosclerotic plaque burden by modulating mTORC1 ubiquitination pathways (284). Meanwhile, histone deacetylase inhibitors that affect acetylation status of mTOR regulators show promise in limiting ischemia-reperfusion injury (285). These therapies focus on modulating key pathways, including mTOR inhibition, regulation of protein modifications, and attenuation of pathological cell death. The table below summarizes critical drugs, their mechanisms of action, and their clinical relevance in cardiovascular diseases (Table 5).

**Table 5:** Therapeutic compounds targeting mTOR signaling or protein modifications in cardiovascular disease models.

Drug category	Representative drugs	Mechanism of action	References
mTOR inhibitors	Sirolimus Everolimus Temsirolimus	Inhibit mTORC1 activity, reduce cardiac hypertrophy, inflammation, and fibrosis, regulate cell proliferation and metabolism	(286)
AMPK activators	Metformin AICAR	Activate AMPK pathway, inhibit mTORC1, restore energy metabolism, reduce phosphorylation imbalance and oxidative stress	(287)
Proteasome inhibitors	Bortezomib Carfilzomib	Inhibit proteasome activity, reduce abnormal protein degradation, stabilize mTOR complex, and inhibit apoptosis and foam cell formation	(288)
E3 ligase modulators	MLN4924 Thalidomide derivatives	Regulate E3 ligase activity, mitigate inflammation-mediated cell death and stress responses	(289)
SUMOylation inhibitors	TAK-981 Anacardic acid	Inhibit SUMOylation, reduce inflammation and phenotypic switching, improve pathological vascular smooth muscle cell behavior	(290)
HDAC inhibitors	Vorinostat Panobinostat Trichostatin A	Regulate acetylation levels, reduce myocardial fibrosis and inflammation, improve metabolic imbalance and cardiac remodeling	(291)
SIRT1 activators	Resveratrol Nicotinamide Mononucleotide	Activate SIRT1 deacetylation, regulate mTOR signaling, enhance antioxidant action and mitochondrial function	(292)
PI3K/AKT inhibitors	LY294002 Wortmannin	Inhibit PI3K/AKT-mTOR pathway, reduce excessive cell proliferation and inflammatory responses	(293)
Antioxidants	N-Acetylcysteine Coenzyme Q10	Reduce oxidative stress, inhibit apoptosis and inflammation through mTOR and downstream pathways	(294)
Iron chelators	Deferoxamine Deferiprone	Reduce iron-dependent oxidative stress and ferroptosis, improve cell survival via mTOR-related mechanisms	(295)
Anti-inflammatory drugs	Colchicine Canakinumab	Reduce inflammatory mediator release, regulate mTOR-associated cell death processes, stabilize lesions	(296)
mTOR activators	MHY1485	Selectively activate mTORC1 to promote cell survival and reduce cardiomyocyte apoptosis	(297)

This table provides a comprehensive overview of the therapeutic landscape targeting protein modifications and mTOR signaling in cardiovascular diseases, emphasizing both established treatments and promising drug candidates.

Notably, several ongoing clinical trials support the translational relevance of targeting mTOR or PTM pathways in cardiovascular diseases (193). For instance, everolimus, an mTORC1 inhibitor, is being evaluated in patients with coronary artery disease via bioresorbable scaffolds (NCT03039751) (298). In addition, the HDAC inhibitor vorinostat, which affects protein acetylation, is under investigation in ischemic heart conditions for its potential to modulate myocardial remodeling (NCT02455034) (299). These studies highlight the clinical momentum toward PTM- and mTOR-based therapies in CVD.

### 6.3 Current limitations and translational challenges

Despite the promising advances in targeting mTOR signaling and PTM pathways for cardiovascular disease treatment, several translational hurdles remain (300). A key concern is the lack of tissue and cell-type specificity of many mTOR inhibitors and PTM modulators, which may lead to off-target effects such as metabolic imbalance, immune suppression, or unintended cell death in non-target tissues (301). Additionally, the intracellular delivery of these agents is often inefficient, particularly for compounds targeting specific protein modifications that require nuclear or organelle-level access (302). Conventional systemic administration may result in suboptimal bioavailability and dose-limiting toxicity. Furthermore, the temporal dynamics of PTMs pose a challenge, as modulating transient or reversible modifications *in vivo* requires precise control (303). Overcoming these obstacles will require improved delivery systems (e.g., nanoparticle carriers or tissue-specific vectors), enhanced selectivity of inhibitors, and deeper understanding of context-dependent mTOR–PTM–cell death networks in various CVD settings (193).

## 7 Conclusion

In conclusion, the mechanistic target of rapamycin (mTOR) signaling pathway and its associated protein modifications are integral to the regulation of various cellular processes such as growth, metabolism, survival, and death (304, 305). These pathways are critically involved in the pathophysiology of cardiovascular diseases (CVDs), including atherosclerosis, heart failure, ischemia-reperfusion injury, and hypertension (306, 307). Dysregulation of mTOR activity, often through aberrant protein modifications such as phosphorylation, ubiquitination, SUMOylation, acetylation, and glycosylation, contributes to maladaptive cellular responses and promotes the progression of these diseases (218). The complex interactions between mTOR signaling and protein modifications play a pivotal role in determining the balance between cell survival and death, influencing processes like apoptosis, autophagy, ferroptosis, and necrosis (308).

Targeting mTOR signaling and its associated protein modifications has emerged as a promising therapeutic strategy in cardiovascular medicine. The development of drugs that modulate mTOR activity, such as mTOR inhibitors, AMPK activators, and proteasome inhibitors, has shown potential in preclinical studies and early clinical trials for restoring homeostasis and reducing pathological cell death. Moreover, pharmacological interventions targeting specific protein modifications, including SUMOylation inhibitors and anti-inflammatory agents, offer new avenues for therapeutic intervention in CVDs. These strategies aim to modulate the intricate cellular mechanisms regulated by mTOR and its modifications, thus offering a refined approach to managing cardiovascular diseases.

However, while significant progress has been made, further research is needed to deepen our understanding of the precise molecular mechanisms through which mTOR signaling and protein modifications contribute to cardiovascular pathology. In particular, the development of more selective and effective drugs that can target specific aspects of mTOR-related pathways and protein modifications is crucial for translating these findings into clinical practice. Additionally, clinical trials evaluating the long-term safety and efficacy of these therapeutic strategies in diverse patient populations will be essential in establishing their therapeutic potential.

Ultimately, the exploration of mTOR signaling and protein modifications represents a promising frontier in cardiovascular research, with the potential to revolutionize the treatment of CVDs. By targeting these molecular pathways, we can offer more personalized and effective therapies, improving patient outcomes and quality of life for individuals suffering from cardiovascular diseases. These disease-specific mechanistic insights emphasize the importance of targeting discrete mTOR-PTM-cell death axes for precision therapy in cardiovascular diseases. Future studies should focus on delineating these pathways in diverse pathologies to optimize therapeutic interventions.
